# The Antibacterial Effect of Cannabigerol toward *Streptococcus mutans* Is Influenced by the Autoinducers 21-CSP and AI-2

**DOI:** 10.3390/biomedicines11030668

**Published:** 2023-02-22

**Authors:** Muna Aqawi, Ronit Vogt Sionov, Michael Friedman, Doron Steinberg

**Affiliations:** 1 The Biofilm Research Laboratory, The Institute of Biomedical and Oral Research (IBOR), The Faculty of Dental Medicine, The Hebrew University of Jerusalem, Jerusalem 9112102, Israel; 2 The Institute of Drug Research, School of Pharmacy, The Hebrew University of Jerusalem, Jerusalem 9112102, Israel

**Keywords:** *Streptococcus mutans*, cannabigerol, quorum sensing, *Vibrio harveyi*, 21-CSP, ComCDE, autoinducer-2

## Abstract

Bacteria can communicate through an intercellular signaling system referred to as quorum sensing (QS). The QS system involves the production of autoinducers that interact with their respective receptors, leading to the induction of specific signal transduction pathways. The QS systems of the oral cariogenic *Streptococcus mutans* regulate the maturation of biofilms and affect its virulent properties. We have previously shown that the non-psychoactive compound cannabigerol (CBG) of the *Cannabis sativa* L. plant has anti-bacterial and anti-biofilm activities towards *S. mutans*. Here we were interested in investigating the effect of the two QS systems ComCDE and LuxS on the susceptibility of *S. mutans* to CBG and the anti-QS activities of CBG. This was assessed by using various *comCDE* and *luxS* mutant strains and complementation with the respective autoinducers, competence stimulating peptide (CSP) and (S)-4,5-dihydroxy-2,3-pentandione (DPD, pre-AI-2). We found that *S. mutans comCDE* knockout strains were more sensitive to the anti-bacterial actions of CBG compared to the WT strain. Exogenously added 21-CSP prevented the anti-bacterial actions caused by CBG on the *ΔcomC*, *ΔcomE* and *ΔluxS* mutants, while having no effect on the susceptibility of the WT and *ΔcomCDE* strains to CBG. Exogenously added DPD increased the susceptibility of WT and *ΔluxS* to CBG. Vice versa, CBG significantly reduced the 21-CSP-induced expression of *comCDE* genes and ComE-regulated genes and suppressed the expression of *luxS* with concomitant reduction in AI-2 production. DPD induced the expression of *comCDE* genes and ComE-regulated genes, and this induction was repressed by CBG. 21-CSP alone had no significant effect on *luxS* gene expression, while *ΔcomCDE* strains showed reduced AI-2 production. In conclusion, our study shows that the susceptibility of *S. mutans* to CBG is affected by the ComCDE and LuxS QS pathways, and CBG is a potential anti-QS compound for *S. mutans*. Additionally, we provide evidence for crosstalk between the ComCDE and LuxS QS systems.

## 1. Introduction

Bacteria can communicate through an intercellular signaling mechanism known as quorum sensing (QS) [1]. Several phenotypes in bacteria are controlled by the QS system. These phenotypes range from basic cell mobility to more complex communal interactions including biofilm formation, production of virulence factors, modulation of metabolic activity, production of extracellular polymeric substance (EPS), acquisition of nutrients, competence of genetic material, resistance to antibiotics and the generation of secondary metabolites [2,3].

The QS systems commonly consist of secreted autoinducers which interact with their respective receptors on the bacteria cell surface, leading to the induction of distinct signal transduction pathways when their concentrations exceed the threshold [4]. This enables the reception of information regarding population and cellular density in the immediate surroundings. The activation of the signal transduction pathways leads to adapted alterations in gene expression [3,5].

The QS pathways have frequently been classified into three categories. The first one encompasses the homologous QS networks in Gram-negative bacteria where the autoinducers are acylated homoserine lactones (AHL) operating in genetic pathways akin to the LuxIR in *Vibrio fischeri* [6,7]. The second category is the two-component signal-transduction pathway of Gram-positive bacteria, with post-translational processed peptides serving as the messengers [8]. The third category is the interspecies communication that relies on autoinducer-2 (AI-2) synthases and the LuxS protein family [9]. AI-2 is a furanosyl borate diester present in numerous Gram-positive and Gram-negative bacteria, and thus considered a universal autoinducer [10].

*Streptococcus mutans* is considered a key pathogen in the human oral cavity [11]. They are Gram-positive, cocci shaped and facultative anaerobic bacteria, and their presence within the dental biofilm is crucial for the formation and maturation of the oral microflora and of the cariogenicity in dental plaque [12]. The ComABCDE QS system of *S. mutans* involves a competence stimulating peptide (CSP)-mediated quorum sensing, which regulates several virulence-associated traits [13]. It is composed of the *comAB* and the *comCDE* operons. The *comC* gene encodes a precursor of CSP, while *comD* is transcribed into a histidine kinase (HK) sensor protein, and *comE* encodes for a cognate response regulator [14,15,16,17]. The CSP propeptide is transported out of the bacteria through the integrated ComAB ABC transporter, and during this transmembrane transport it is processed into 21-CSP. The secreted 21-CSP is, in turn, converted to the mature 18-CSP autoinducer by the extracellular protease SepM, which then binds to its cognate membrane receptor ComD [18]. Upon activation of ComD, it phosphorylates and activates its cognate response regulator ComE. This causes the induction of genes involved in the regulation of biofilm formation and genetic competence (e.g., *comX/sigX* that induces the expression of late competence genes), as well as the induction of the *nlmA-D* bacteriocin genes [13,15,19].

*S. mutans* also possesses a LuxS QS-signaling pathway that mediates interspecies communication [9,20]. The *luxS* gene encodes for an enzyme involved in the production of (S)-4,5-dihydroxy-2,3-pentandione (DPD), which is a precursor of AI-2 furanones. When the extracellular AI-2 level exceeds a threshold, it induces a signal cascade that leads to large alterations in gene expression, among others, the induction of virulence genes [17,21,22].

The non-psychoactive *Cannabis* compound, cannabigerol (CBG), is known to be a precursor molecule for Δ^9^-tetrahydrocannabinol (Δ^9^-THC) and cannabidiol (CBD) [23,24]. In certain industrial hemps, it can also be found at elevated levels [25]. CBG has been shown to exert anti-nociceptive, anti-inflammatory and anti-oxidative effects and was shown to be beneficial when used to treat inflammatory bowel disease and neurological disorders, including Parkinson’s disease, multiple sclerosis and Huntington’s disease [24,26,27,28,29]. From a microbiological point of view, CBG has been shown to have an antimicrobial effect against *Streptococcus mutans*, *Streptococcus sanguis*, *Streptococcus sobrinus*, *Streptococcus salivarius*, *Neisseria gonorrhoeae*, *Staphylococcus aureus* and methicillin-resistant *Staphylococcus aureus* (MRSA) [30,31,32,33]. Moreover, it exerts anti-biofilm activities against MRSA and *Streptococcus mutans* [30,31,33,34].

We have previously shown that CBG exerts an anti-QS activity towards *Vibrio harveyi* [35]. It reduced the QS-regulated bioluminescence, motility and biofilm formation of *Vibrio harveyi*. These effects are, among others, caused by an increase in LuxO expression and activity, with a concomitant downregulation of the LuxR gene [35]. In light of these findings, we were interested in studying whether CBG also had an anti-QS effect on *S. mutans*. This trait is especially important as there is a growing interest for compounds with anti-QS activities [36]. We specifically focused our study on the ComABCDE and LuxS QS systems. Surprisingly, we observed that *comCDE* mutant strains were more sensitive than WT to the anti-bacterial effect of CBG, and the exogenously added 21-CSP antagonized it, suggesting that the ComCDE QS provides pro-survival signals. In contrast, DPD (pre-AI-2) strengthens the anti-bacterial effect of CBG. Thus, the QS pathways may modulate the susceptibility of *S. mutans* to CBG. CBG itself prevented the expression of *luxS*, *comCDE* and ComE-regulated genes, indicating that this compound possesses anti-QS properties. Additionally, we provide evidence that there is a crosstalk between the LuxS and ComCDE systems in *S. mutans*, where DPD (pre-AI-2) induced the expression of *comCDE* and ComE-regulated genes, and a lack of *comCDE* resulted in reduced AI-2 production. The results obtained from this study provide novel insights into the molecular pathways regulating the susceptibility of *S. mutans* to CBG and adds another layer to the anti-bacterial action mechanism of CBG.

## 2. Materials and Methods

### 2.1. Materials

The hemp isolate, cannabigerol (CBG) (95% purity) was purchased from NC Labs (Czech Republic) and dissolved in ethanol at a concentration of 10 mg/mL. The control samples were incubated with respective dilutions of ethanol (0.0075–0.05%). (S)-4,5-Dihydroxy-2,3-pentandione (DPD, a precursor of AI-2) was obtained from OMM Scientific Inc. (Dallas, TX, USA) and dissolved at a concentration of 0.55 mg/mL in double distilled water (DDW). The 21-CSP (NH2-SGSLSTFFRLFNRSFTQALGK-COOH) was synthesized by Syntezza Bioscience Ltd., Jerusalem, Israel, with a purity grade of >95%, and dissolved in DDW at a concentration of 100 µg/mL.

### 2.2. Bacterial Strains, Culture Conditions and End Point Planktonic Growth Assay

A starter culture of *S. mutans* UA159 (WT), *S. mutans ΔluxS* (TW26), *S. mutans ΔcomC*, *S. mutans ΔcomE* and *S. mutans ΔcomCDE* were incubated in brain heart infusion broth (BHI, Acumedia, Lansing, Michigan, USA) for 20–24 h at 37 °C in the presence of 5% CO_2_ until an OD_600nm_ of 1.0–1.2 was obtained [32]. The *S. mutans ΔluxS* (TW26) was generously provided by Professor Robert Burne (University of Florida, Gainesville, FL, USA) [37]. *S. mutans ΔcomC*, *S. mutans ΔcomE* and *S. mutans ΔcomCDE* mutants were kindly provided by Professor Howard Kuramitsu (State University of New York, Albany, NY, USA) [38]. For the planktonic growth assays, the bacterial cultures were adjusted to an OD_600nm_ of 0.1 in fresh BHI and exposed to various concentrations of CBG (0.75, 1.25, 2.5 and 5 µg/mL) or respective concentrations of ethanol (0.0075–0.05%) and seeded in a volume of 200 µL in a sterile tissue-grade transparent flat-bottom 96-well microplate (Corning, Incorporated, Kennebunk, ME, USA). Untreated bacteria served as an additional control. After a 24 h incubation at 37 °C, the absorbance was measured at 595 nm by a Tecan M200 infinite microplate reader (Tecan Trading AG, Männedorf, Switzerland). The bacterial viability is expressed as percentage in comparison to control sample according to the following formula: % Viability = (OD_treated sample_)/(OD_control sample_) × 100.

### 2.3. Growth Kinetics Studies

The influence of DPD (pre-AI-2)/21-CSP on planktonic growing *S. mutans* strains was examined by performing kinetics studies. Overnight cultures of *S. mutans* UA159, *S. mutans ΔluxS* (TW26), *S. mutans ΔcomC*, *S. mutans ΔcomE* and *S. mutans ΔcomCDE* bacteria were made to an OD_600nm_ of 0.1 in fresh BHI medium and exposed to varying concentrations of CBG (0.75–5 µg/mL) in the presence or absence of 21-CSP (1 µg/mL) or DPD (5 µM). The bacteria were then cultivated in a transparent flat-bottom 96-well microplate (Corning) at a volume of 200 µL, and the bacterial growth (OD_595nm_) was measured every 30 min for 20 h in a Tecan M200 microplate reader (Tecan Trading AG, Männedorf, Switzerland) at 37 °C [32].

### 2.4. Investigation of AI-2 Production

AI-2 production by *S. mutans* under different concentrations of CBG was determined by using *Vibrio harveyi* reporter strain MM77 in a bioluminescence assay, similar to the method of Shemesh et al. [39]. MM77 was kindly provided by Professor Bonnie Bassler (Princeton University, Princeton, NJ, USA) [40]. The wild-type (WT) and mutant *S. mutans* strains (OD_595nm_ = 0.1) were allowed to grow in BHI media for 4 h in order to reach the early logarithmic growth phase in the absence or presence of varying concentrations of CBG (0.75–5 µg/mL) and/or 21-CSP (1 µg/mL). The condition medium (CM) was collected after removing the bacteria by centrifugation and was passed through a 0.22 µm pore size sterile PVDF filter (Millex-GV, Merck, Darmstadt, Germany). The CMs were stored at −20 °C until future use. Bioluminescence assay was performed as described previously by Aqawi et al. [35]. Briefly, *V. harveyi* MM77 (luxLM::Tn5, luxS::CmR; lacking both AI-1 and AI-2) were grown for 20–24 h at 30 °C in AB medium with constant shaking until reaching stationary phase (OD_600nm_ = 0.7). The MM77 bacteria at a starting OD of 0.035 were incubated at 30 °C in AB medium containing 10% (*v*/*v*) *S. mutans* CM in white optical 96-well flat-bottomed plates (μCLEAR CELLSTAR, Greiner Bio-One, Frickenhausen, Germany). MM77 bacteria incubated in 10% fresh BHI medium served as a negative control and MM77 incubated with 5 µM DPD served as a positive control. Luminescence and absorbance (OD_595nm_) were measured in parallel each 30 min for 48 h using the Tecan M200 infinite microplate reader (Tecan Trading AG, Switzerland). Luminescence values were normalized by OD values of each measurement time point to adjust for any differences in *V. harveyi* growth rates. The resulting luminescence was then divided on the OD_600nm_ of the various *S. mutans* cultures used for collecting the CMs, to correct for different numbers of bacteria. The data are expressed as the ratio of luminescence/OD, and calculations for the area under the curve (AUC) were performed.

### 2.5. RNA Isolation

Overnight cultures of the various *S. mutans* strains were adjusted to OD_600nm_ = 0.1 in BHI and incubated in the absence or presence of 1.25 µg/mL CBG and/or 21-CSP (1 µg/mL). After a 4 h incubation at 37 °C, the bacteria were resuspended in 1 mL RNA protect reagent (Qiagen, Hilden, Germany) for 5 min. Following centrifugation (1500× *g*, 15 min, 4 °C), the pellets were stored at −80 °C. On the day of RNA extraction, the pellets were resuspended in 350 µL RLT lysis buffer (Qiagen, Hilden, Germany), placed into microcentrifuge tubes containing 1 mm glass beads followed by beating 3 times at a speed of 4.5 m/s for 45 s at 5 min intervals using the Bio 101 FastPrep FP120 cell disruption system (Savant Instruments, Inc., Holbrook, NY, USA). The samples were further processed for RNA isolation using the RNeasy MINI kit (Qiagen, Hilden, Germany) according to the manufacturer’s instructions including an on-column DNase digestion step [35]. The RNA concentration and purity were analyzed by a Nanodrop (Nanovue, GE Healthcare Life Sciences, Buckinghamshire, UK). Samples that had an OD_260_/OD_280_ ratio of 1.8–2 and an OD_260_/OD_230_ ratio above 2 were used for cDNA synthesis. The RNA was reverse transcribed into cDNA using the qScript cDNA synthesis kit (QuantaBio, Beverly, MA, USA) [35].

### 2.6. Quantitative Real-Time PCR

Quantitative real-time PCR was performed as described by Aqawi et al. [35]. Each reaction was prepared by mixing 10 ng cDNA with 300 nM of respective forward and reverse primers (Supplementary Table S1) and Power Sybr Green PCR Master mix (Applied Biosystems, Warrington UK). Triplicates were done for each gene of each sample. The amplification was done in a Bio-Rad CFX Connect Real-time system using the Bio-Rad CFX Maestro program with 40 cycles of 15 sec at 95 °C and 1 min at 60 °C. The 16S rRNA was used as a housekeeping gene and the changes in gene expression were calculated using the 2^−ΔΔCt^ method. The control was set to 1 for each gene, and gene expression is presented as relative values.

### 2.7. Statistical Analysis

Each experiment was conducted in triplicate. The student’s *t*-test was used for statistical analysis of the collected data. A *p* value of less than 0.05 in comparison to control was considered statistically significant.

## 3. Results

### 3.1. Streptococcus Mutans Strains Deficient in the comCDE QS System Showed Increased Sensitivity to CBG

**Figure 1:**
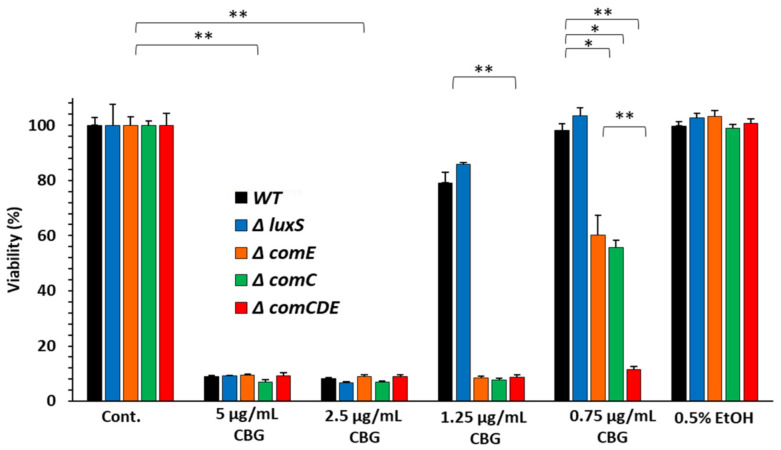
*Streptococcus mutans* strains deficient in the ComCDE QS system showed increased sensitivity to CBG. The viability of different *S. mutans* strains (WT, *ΔluxS*, *ΔcomC*, *ΔcomE* and *ΔcomCDE*) after a 24 h incubation with increasing doses of CBG (0.75–5 µg/mL) as measured by OD_595nm_. *n* = 3; * *p* < 0.05; ** *p* < 0.01.

To explore the effect of quorum sensing on the susceptibility of *S. mutans* to the anti-bacterial activity of CBG, *S. mutans* UA159 (WT) and different QS-knockout strains (*S. mutans ΔluxS*, *S. mutans ΔcomC*, *S. mutans ΔcomE* and *S. mutans ΔcomCDE*) were exposed to increasing concentrations of CBG (0.75–5 µg/mL) or the respective ethanol dilutions and the OD_595nm_ was monitored after a 24 h incubation (Figure 1). Untreated *S. mutans* from each strain served as another control. We observed that the different *comCDE*-knockout strains showed higher susceptibility to CBG than the WT strain, while the *ΔluxS* strain showed similar susceptibility to CBG as the WT (Figure 1), suggesting that the ComCDE signaling pathway may promote bacterial survival. The MIC of CBG on the WT and *ΔluxS* strains was found to be 2.5 µg/mL, while that of the *ΔcomC* and *ΔcomE* strains was 1.25 µg/mL. The triple *ΔcomCDE* mutant strain showed an even higher susceptibility than each of the single knockouts, with a MIC of 0.75 µg/mL. Altogether, these data suggest that the anti-bacterial effect of CBG is influenced by the ComCDE quorum sensing system.

### 3.2. 21-CSP Prevented the Anti-Bacterial Effect of CBG on the ΔcomC and ΔcomE Strains, but Not on ΔcomCDE

**Figure 2:**
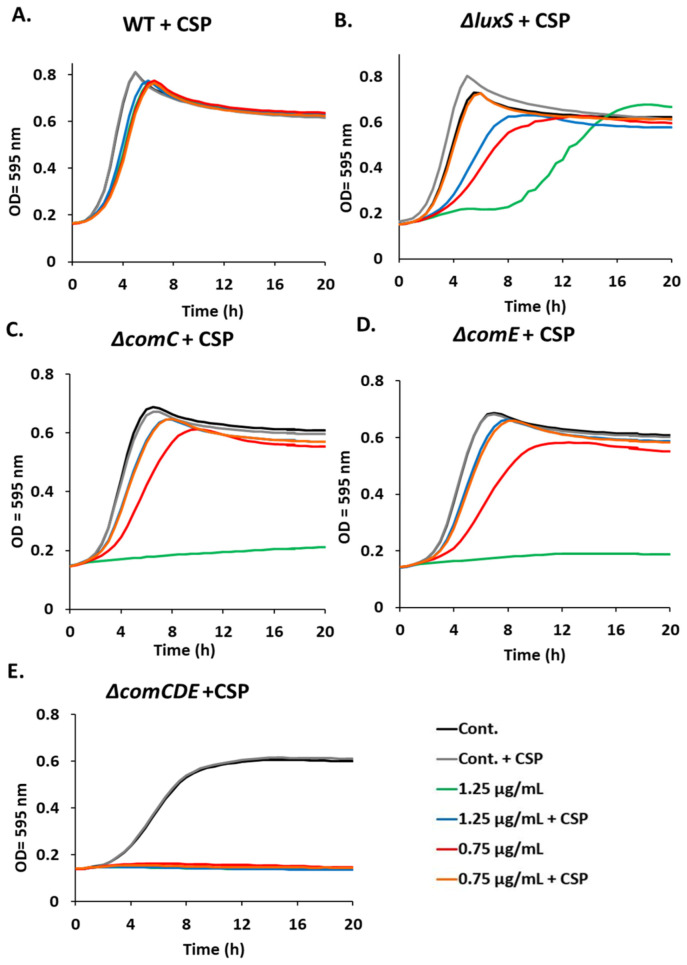
21-CSP prevented the anti-bacterial effect of CBG on the *ΔcomC* and *ΔcomE* strains. (**A**–**E**). Kinetic studies of the planktonic growth of the various *S. mutans* strains incubated in the absence or presence of CBG (1.25 µg/mL) and/or 21-CSP (1 µg/mL) with an initial OD_600nm_ of 0.1. (**A**). WT; (**B**). *ΔluxS*; (**C**). *ΔcomC*; (**D**). *ΔcomE*; and (**E**). *ΔcomCDE*. *n* = 3.

To test the effect of the 21-CSP signal on the anti-bacterial effect of CBG on the different *S. mutans* mutant strains, the different strains were exposed to CBG (0.75–5 µg/mL) in the absence or presence of 1 µg/mL of 21-CSP, and the bacterial growth was analyzed in a kinetic study by measuring changes in the optical density. 21-CSP alone caused a minor increase in the bacterial growth of the WT strain (Figure 2A) and did not affect the bacterial growth in the presence of CBG at the tested concentrations (0.75–5 µg/mL) (Figure 2A and Figure S1A). 21-CSP significantly prevented the delay in the log phase growth of the *ΔluxS* strain caused by 1.25 µg/mL CBG (Figure 2B). Notably, 21-CSP antagonized the anti-bacterial effect induced by 1.25 µg/mL CBG on *ΔcomC* (Figure 2C) and *ΔcomE* (Figure 2D), suggesting that 21-CSP induces a cell survival response that opposes the CBG anti-bacterial effects. The anti-bacterial effect of CBG against the *ΔcomCDE* mutant remained unchanged despite the presence of 21-CSP (Figure 2E). Since the *ΔcomE* mutant responded to 21-CSP, this indicates that the survival response induced by 21-CSP is mediated by a different pathway than the classical ComCDE cascade. However, since 21-CSP had no effect on the triple *ΔcomCDE* mutant (Figure 2E), it is likely that the signal depends on the ComD receptor. It is noteworthy that at the higher CBG concentrations (2.5 and 5 µg/mL), 21-CSP exerted no effect on any of the *S. mutans* mutant strains (Figure S1). This might be due to the activation of cell death pathways in the bacteria upon higher concentrations.

### 3.3. DPD Increased the Susceptibility of WT and ΔluxS, but Not of ΔcomC or ΔcomE, to CBG

**Figure 3:**
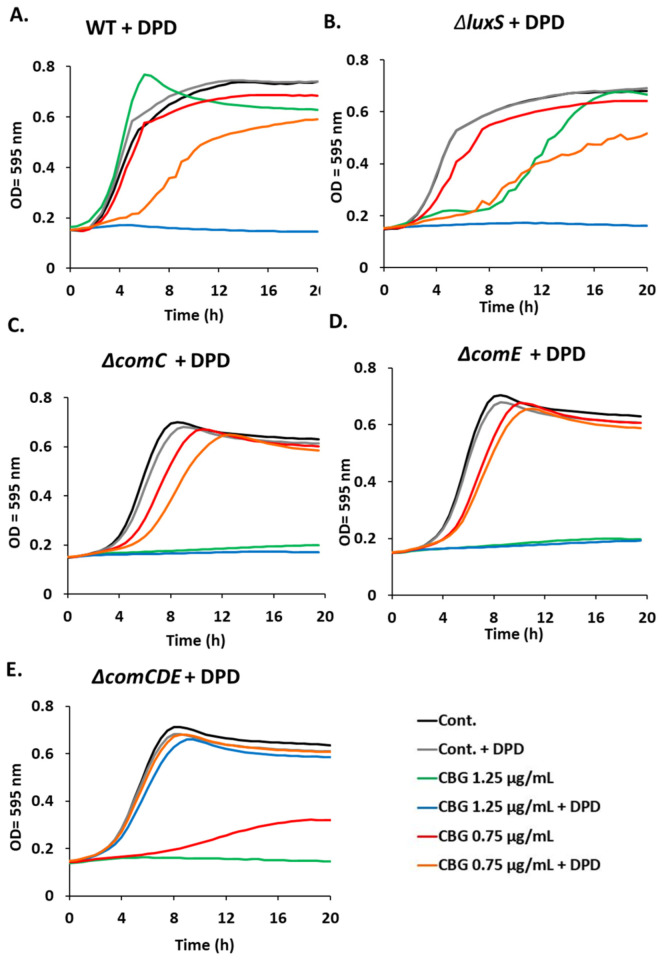
DPD increased the susceptibility of WT and *ΔluxS* strains, but not of the *ΔcomC* and *ΔcomE* strains to CBG. (**A**–**E**). Kinetic studies of the planktonic growth of the various *S. mutans* strains incubated in the absence or presence of CBG (1.25 µg/mL) and/or DPD (pre-AI-2) (5 µM) with an initial OD_600nm_ of 0.1. (**A**). WT; (**B**). *ΔluxS*; (**C**). *ΔcomC*; (**D**). *ΔcomE*; and (**E**). *ΔcomCDE*. *n* = 3.

To examine the effect of the AI-2 signal on the anti-bacterial effect of CBG on the different *S. mutans* mutant strains, the strains were exposed to CBG (0.75–5 µg/mL) in the absence or presence of 5 µM of DPD (pre-AI-2), and the bacterial growth was analyzed in a kinetic study by measuring changes in the OD. We found that AI-2 increased the susceptibility of the WT and the *ΔluxS* strains to the anti-bacterial effect of CBG (Figure 3A,B). The combined treatment of DPD with 1.25 µg/mL CBG completely prevented the bacterial growth of these two strains (Figure 3A,B). At 0.75 µg/mL CBG, DPD delayed the log growth phase, and the bacteria did not reach the OD of the control bacteria even after a 20 h incubation (Figure 3A,B). DPD did not significantly affect the sensitivity of the *ΔcomC* (Figure 3C) and *ΔcomE* (Figure 3D) to CBG, and surprisingly, DPD actually inhibited the anti-bacterial activity of 1.25 and 0.75 µg/mL CBG on the *ΔcomCDE* strain (Figure 3E). DPD had no observable effects on the bacterial growth when using higher CBG concentrations (2.5 and 5 µg/mL) (Figure S2), which might be due to the already strong anti-bacterial activity at these concentrations.

### 3.4. CBG Antagonizes the 21-CSP-Induced Gene Expression of nlmA, nlmB and nlmC/cipB

**Figure 4:**
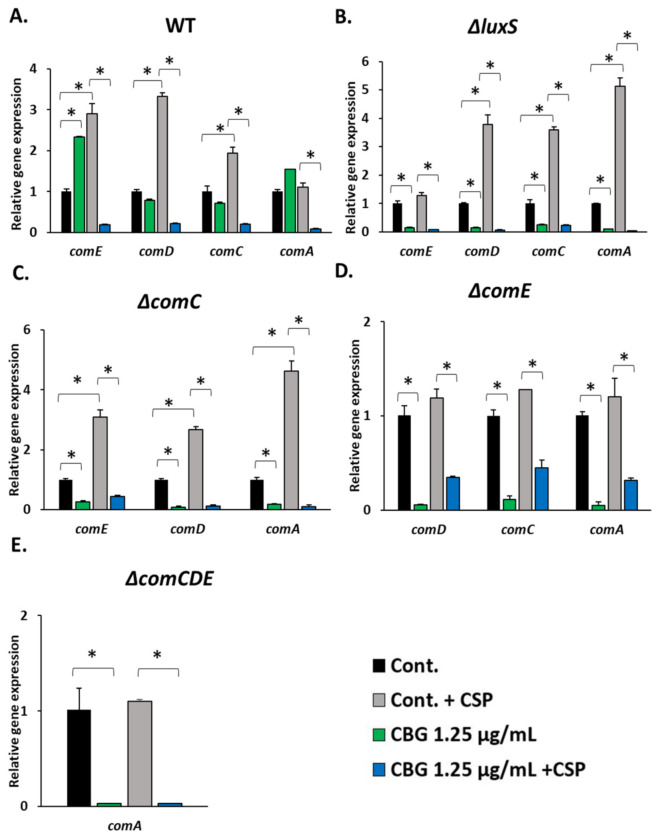
CBG antagonizes the 21-CSP-induced gene expression of *comCDE* genes. (**A**–**E**). Expression of *comCDE* genes in various *S. mutans* strains incubated in the absence or presence of 1.25 µg/mL CBG and/or 1 µg/mL 21-CSP for 4 h in comparison to control as determined by real-time PCR using respective primers. (**A**). WT; (**B**). *ΔluxS*; (**C**). *ΔcomC*; (**D**). *ΔcomE*; and (**E**). *ΔcomCDE*. *n* = 3; * *p* < 0.05.

Next, we were interested in studying the effect of CBG on *comCDE* gene expression and the expression of genes induced by 21-CSP. The different *S. mutant* strains were exposed to 1.25 µg/mL CBG in the absence or presence of 1 µg/mL 21-CSP for 4 h, and then the changes in gene expression were studied by real-time qPCR. While CBG alone had some variable effects on *comCDE* gene expression in the WT strain, it reduced their expression in the *ΔluxS*, *ΔcomC* and *ΔcomE* strains (Figure 4A–E). The *comA* gene expression was also reduced by CBG in these three strains as well as in the *ΔcomCDE* strain (Figure 4A–E), suggesting that its suppression by CBG is independent of the ComCDE QS cascade. The addition of 21-CSP led to the induction of *comCDE* genes in the WT, *ΔluxS* and *ΔcomC* strains, while no effect was seen on the *ΔcomE* and *ΔcomCDE* strains (Figure 4A–E), which confirms that this induction is dependent on the regulatory ComE transcription factor. Notably, CBG strongly reduced the 21-CSP-induced expression of *comCDE* genes (Figure 4A–E).

**Figure 5:**
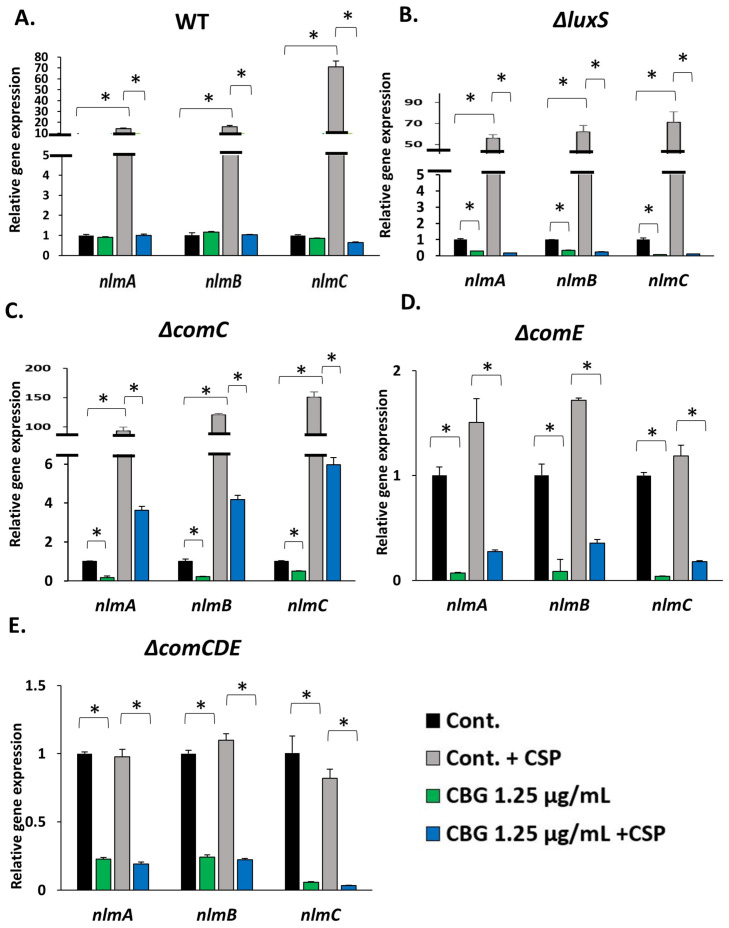
CBG antagonized the 21-CSP-induced gene expression of ComE-regulated genes. (**A**–**E**)**.** Expression of ComE-regulated genes in various *S. mutans* strains incubated in the absence or presence of 1.25 µg/mL CBG and/or 1 µg/mL 21-CSP for 4 h in comparison to control as determined by real-time PCR using respective primers. (**A**). WT; (**B**). *ΔluxS*; (**C**). *ΔcomC*; (**D**). *ΔcomE*; and (**E**). *ΔcomCDE*. *n* = 3; * *p* < 0.05.

To further confirm that CBG has an inhibitory effect on the ComCDE QS system, its effect on ComE-regulated genes was studied. CBG at 1.25 µg/mL had only a mild inhibitory effect on the expression of the *nlmA*, *nlmB* and *nlmC* genes in the WT strain (Figure 5A–E), and it consistently reduced the expression of these genes in the four mutant bacterial strains (Figure 5A–E). CBG strongly inhibited the 21-CSP-induced expression of these genes in the WT, *ΔluxS* and *ΔcomC* strains (Figure 5A–E). As expected, 21-CSP did not induce the expression of the ComCDE-regulated genes in the *ΔcomE* and *ΔcomCDE* strains (Figure 5A–E). Since the expression of *nlmA*, *nlmB* and *nlmC* genes were repressed by CBG in the *ΔcomE* and *ΔcomCDE* strains, this indicates that the repression is independent of ComE, or the CBG-mediated suppression is downstream to ComE.

### 3.5. DPD Induced the Expression of comCDE and ComE-Regulated Genes, While the 21-CSP Did Not Affect the Gene Expression of luxS

**Figure 6:**
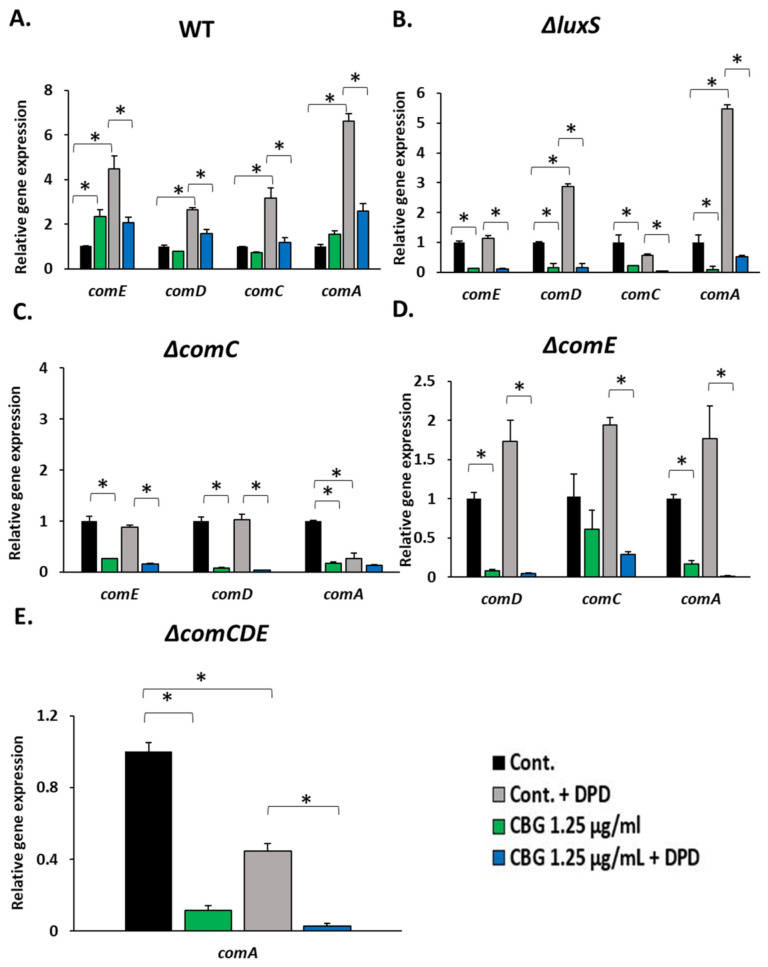
DPD induced the expression of *comCDE* and *comA* genes. (**A**–**E**). Expression of *comCDE* and *comA* genes in various *S. mutans* strains incubated in the absence or presence of 1.25 µg/mL CBG and/or 5 µM DPD (pre-AI-2) for 4 h in comparison to control as determined by real-time PCR using respective primers. (**A**). WT; (**B**). *ΔluxS*; (**C**). *ΔcomC*; (**D**). *ΔcomE*; and (**E**). *ΔcomCDE*. *n* = 3; * *p* < 0.05.

**Figure 7:**
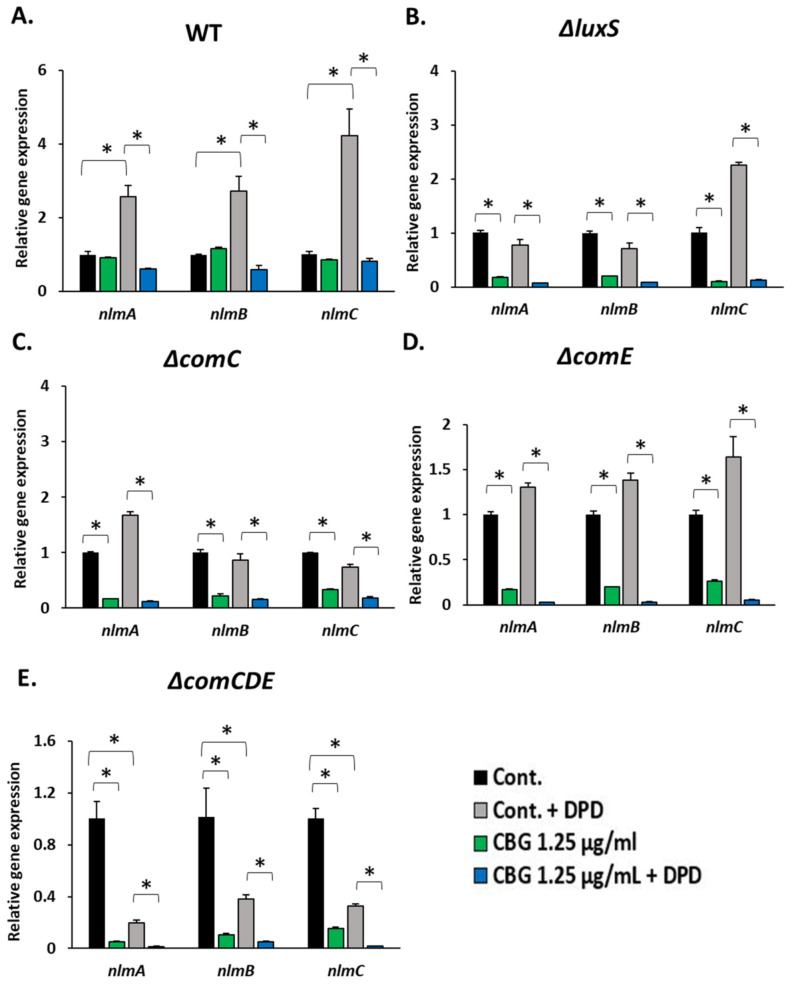
DPD induced the expression of the ComE-regulated genes. (**A**–**E**). Expression of ComE-regulated genes in various *S. mutans* strains incubated in the absence or presence of 1.25 µg/mL CBG and/or 5 µM DPD (pre-AI-2) for 4 h in comparison to control as determined by real-time PCR using respective primers. (**A**). WT; (**B**). *ΔluxS*; (**C**). *ΔcomC*; (**D**). *ΔcomE*; and (**E**). *ΔcomCDE*. *n* = 3; * *p* < 0.05.

**Figure 8:**
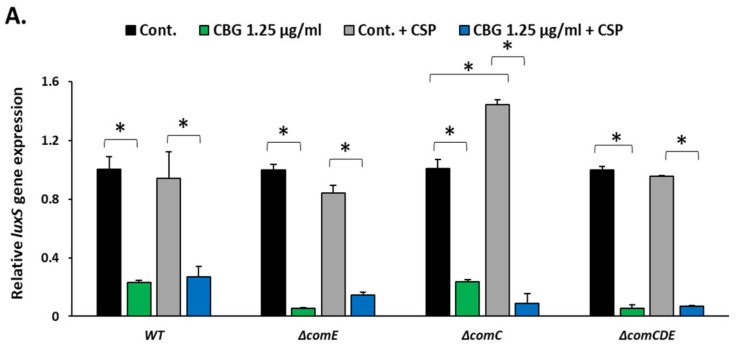

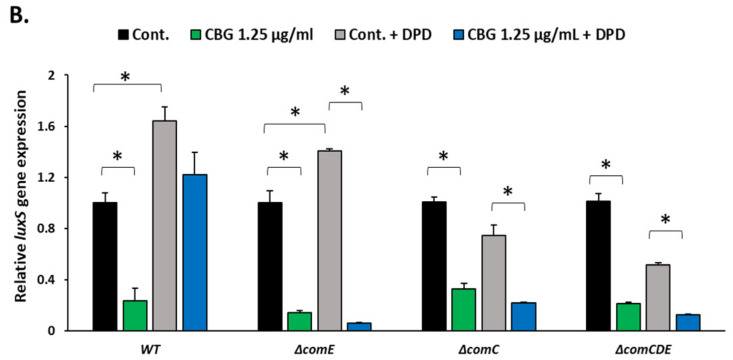
Effect of CBG on *luxS* gene expression. (**A**). Expression of *luxS* gene in various *S. mutans* strains incubated in the absence or presence of 1.25 µg/mL CBG and/or 1 µg/mL 21-CSP for 4 h in comparison to control as determined by real-time PCR using respective primers. (**B**)**.** Expression of *luxS* gene in various *S. mutans* strains incubated in the absence or presence of 1.25 µg/mL CBG and/or 5 µM DPD (pre-AI-2) for 4 h in comparison to control as determined by real-time PCR using respective primers. *n* = 3; * *p* < 0.05.

There are some indications in the literature that suggest a crosstalk between the LuxS and ComCDE QS systems, but so far substantial data are lacking. We therefore decided to use our system to further investigate this possible crosstalk. We first studied the effect of 5 µM of DPD (pre-AI-2) on the expression of the *comCDE* and *comA* gene expression following a 4 h incubation. We found that DPD induced the expression of both the *comCDE* and *comA* genes in the WT strain with a stronger effect on *comA* expression (Figure 6A). This is reflected in the enhanced expression of the *nlmA*, *nlmB* and *nlmC* genes (Figure 7A). In the *ΔluxS* strain, DPD significantly induced the expression of the *comD*, *comA* and *nlmC* genes (Figure 6B and Figure 7B). The *comCDE* genes and ComE-regulated genes were not induced by DPD in the *ΔcomC* strain (Figure 6C and Figure 7C), while a 1.5–2-fold induction was observed in the *ΔcomE* strain in the presence of AI-2 (Figure 6D), suggesting a dependency on CSP, and involvement of both ComE-dependent and ComE-independent pathways. Surprisingly, DPD inhibited the expression of the ComE-regulated genes in the *ΔcomCDE* strain (Figure 7E), suggesting for an AI-2 induced repression in the absence of ComCDE. CBG inhibited the DPD-induced gene expression in the WT, *ΔluxS* and *ΔcomE* strains (Figure 6A,B,D). Notably, 21-CSP and DPD did not affect *luxS* expression in either of the mutant strains (Figure 8A,B), while CBG significantly reduced the *luxS* gene expression in all conditions (Figure 8A,B).

### 3.6. CBG Significantly Reduced the Production of AI-2

**Figure 9:**
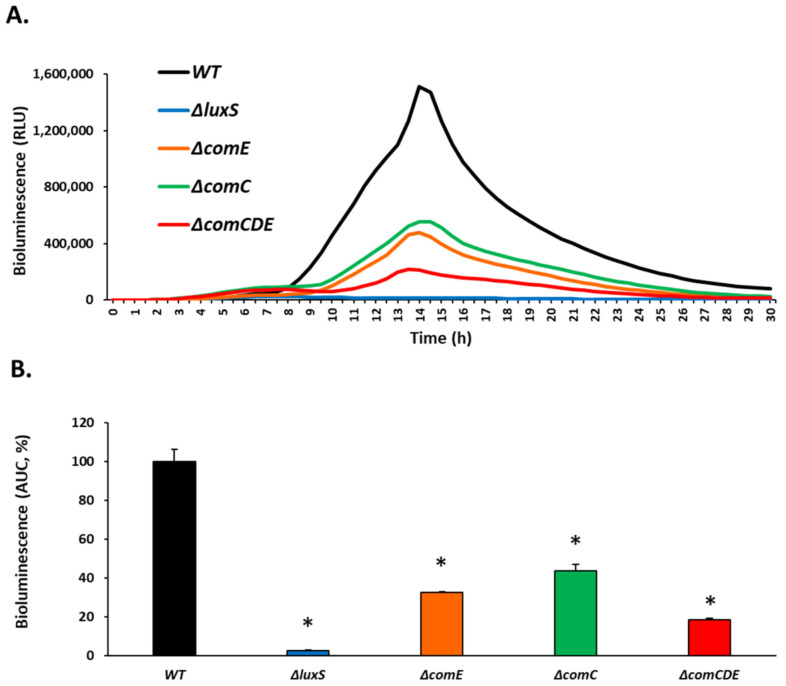
Measuring the AI-2 content in the condition medium (CM) of the various *S. mutans* strains. (**A**). The bioluminescence induced in MM77 *Vibrio harveyi* strain by the condition medium collected from the various *S. mutans* strains (WT, *ΔluxS*, *ΔcomC*, *ΔcomE* and *ΔcomCDE*) was measured for 30 h. The bioluminescence was normalized to differences in bacterial growth by concomitantly measuring the optical density at 595 nm. *n* = 3. (**B**). The relative bioluminescence calculated as the area under the curve (AUC) of the graph presented in (**A**). *n* = 3; * *p* < 0.05.

**Figure 10:**
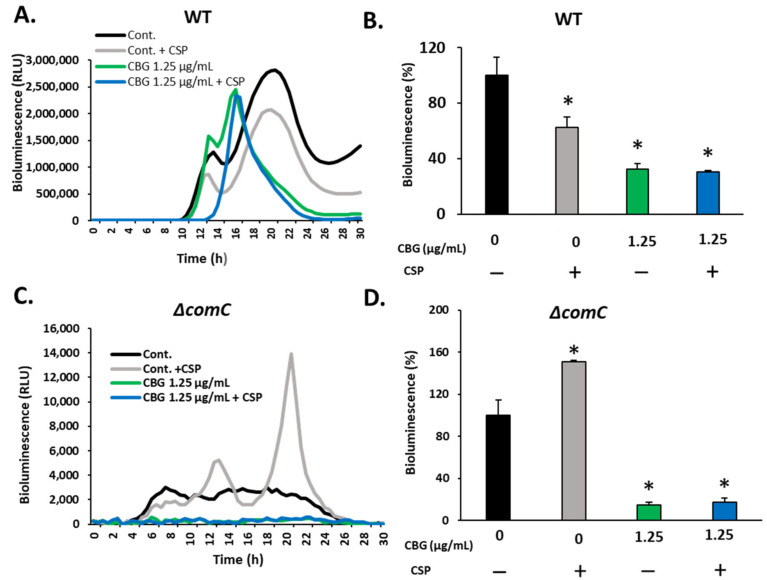

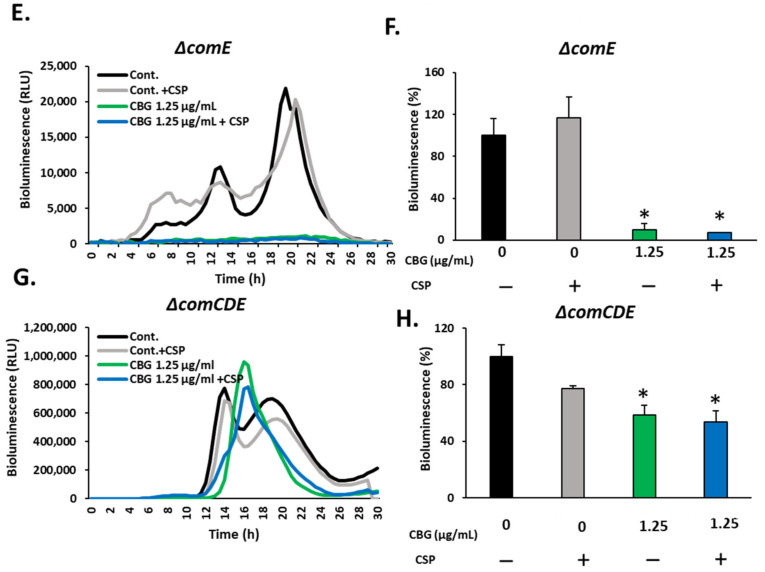
CBG significantly reduced the production of AI-2 by *S. mutans*. (**A**,**C**,**E**,**G**). The relative bioluminescence induced in the MM77 *Vibrio harveyi* strain when incubated with 10% of CM collected from the various S. *mutans* strains that have been exposed to 1.25 µg/mL CBG and/or 1 µg/mL 21-CSP for 4 h. The bioluminescence was normalized for differences in bacterial growth by concomitantly measuring the optical density at 595 nm. *n* = 3; * *p* < 0.05. (**A**). WT; (**C**). *ΔcomC*; (**E**). *ΔcomE*; (**G**). *ΔcomCDE*. *n* = 3. (**B**,**D**,**F**,**H**). The relative bioluminescence as determined by the area under the curve (AUC) of the graph presented in (**A**,**C**,**E**,**G)**, respectively. *n* = 3; * *p* < 0.05.

To study the effect of 21-CSP and/or CBG on AI-2 production by *S. mutans*, the various strains were incubated with different concentrations of CBG for 4 h in the absence or presence of 1 µg/mL 21-CSP, and then the *V. harveyi* MM77 reporter strain was exposed to 10% of the collected conditioned medium (CM) and the luminescence emission was monitored in a kinetic assay. The OD_600nm_ of the *S. mutans* samples were measured at the time of CM collection, and the bioluminescence obtained was divided by this OD to correct for the different cell densities. As expected [41], the double *luxM*, *luxS* null MM77 mutant lacking both AI-1 and AI-2 did not emit bioluminescence by itself (Figure S3A,B), and as a control, exogenously added DPD (pre-AI-2) induced a strong bioluminescence response (Figure S3A,B). No difference was seen in the DPD (pre-AI-2)-induced bioluminescence when 21-CSP was added as a control (Figure S3A,B), indicating that 21-CSP did not interfere with the bioassay. Additionally, the presence of 0.125 µg/mL CBG did not interfere with the bioassay (Figure S4). BHI was added as a control and caused no bioluminescence of the MM77 cells (Figure S3A,B). Different *S. mutans* strains showed different basal bioluminescence. In comparison to WT, the *ΔcomE* strain exhibited a 68 ± 0.5% reduction in the bioluminescence, while *ΔcomC* and *ΔcomCDE* exhibited a reduction of 56 ± 3% and 82 ± 0.5%, respectively (Figure 9), suggesting that the ComCDE QS pathway may affect genes involved in the secretion of AI-2. Due to the absence of the AI-2-producing gene *luxS* in the *ΔluxS* strain, no bioluminescence was emitted (Figure 9). 21-CSP reduced the AI-2 production in the WT strain by 37 ± 4% (Figure 10A,B), while significantly increasing the expression in the *ΔcomC* strain by 50 ± 1.6% (Figure 10C,D). 21-CSP had no significant effect on the AI-2 production in the *ΔcomE* and *ΔcomCDE* strains (Figure 10E–H), suggesting that the 21-CSP-induced AI-2 expression depends on the ComDE signal transduction pathway. CBG at 1.25 µg/mL reduced the AI-2 production in WT, *ΔcomC* and *ΔcomE* strains (Figure 10A–F), while barely having any effect on the *ΔcomCDE* strain (Figure 10G,H), suggesting that ComD might be involved in the CBG-mediated downregulation of AI-2 production. Further studies are required to prove this hypothesis.

## 4. Discussion

Targeting QS pathways has gained popularity during the last several years because of its many applications within medicine and agriculture [42,43,44]. By interrupting the communication between bacteria, QS-regulated virulence factors can be suppressed [45]. This has important clinical implications for treating infectious diseases, especially those caused by antibiotic resistant strains [46].

Several plant species and even bacteria themselves have been found to produce anti-QS substances [44,47,48]. A classic example is the halogenated furanones produced by the Australian red alga (*Delisea pulchra*), which inhibit the QS system of the marine bacterium *Serratia liquefaciens* [49] as well as other QS systems in Gram-negative bacteria [50]. The anti-QS compounds can act at several levels [44,51]: (i) They might prevent the binding of the autoinducer to its receptor (e.g., phytol binds to CviR of *Chromobacterium violaceum*) [52]. (ii) They might inhibit the phosphorelay induced by the autoinducer receptor by either preventing the phosphorylation of downstream mediators or by repressing the expression of the receptor (e.g., cinnamaldehyde represses the expression of LasB, RhlA and PqsA in *Pseudomonas aeruginosa*) [53]. (iii) They might interact with the transcriptional regulator, thereby preventing the activation of the target genes (e.g., LasR antagonists) [35,54,55]. (iv) They may prevent the synthesis of the autoinducer (e.g., carvacrol inhibits LasI AHL synthetase of *Pseudomonas aeruginosa*) [56]. (v) Finally, they might cause the degradation of the autoinducer (e.g., AHL-degradation enzymes of *Rhodosporidium* and *Rhodococcus* species) [44,57].

Various plant species including the *Cannabis sativa* L. plant have received much attention due to their therapeutic potential against microorganisms [24,58]. CBG, a non-psychoactive cannabis constituent was found to exert anti-quorum sensing properties against *Vibrio harveyi* [35]. With regards to *S. mutans*, CBG was found to exert anti-bacterial and anti-biofilm properties [32,33]. CBG exerts a bacteriostatic effect on *S. mutans* that is affected by the initial bacterial cell density. It also affects properties of the membrane and structure, causing immediate membrane hyperpolarization, increase in the membrane permeability, reduction in the fluidity of the membrane and accumulation of mesosome-like membrane structures [32]. Moreover, CBG prevented the decrease in pH that is usually caused by *S. mutans*, thereby preventing is cariogenic property [32]. In addition to the anti-bacterial effects of CBG, it also demonstrated anti-biofilm activities. CBG directly inhibited the formation of biofilms by acting as an anti-bacterial agent, and indirectly by acting on metabolic pathways that regulate the formation of biofilms. CBG also reduced essential biofilm-regulating gene expression, prevented the production of EPS and caused an induction of reactive oxygen species (ROS) production [33]. Due to the involvement of QS in biofilm formation, we have here investigated the anti-QS activity of CBG on *S. mutans*.

**Figure 11:**
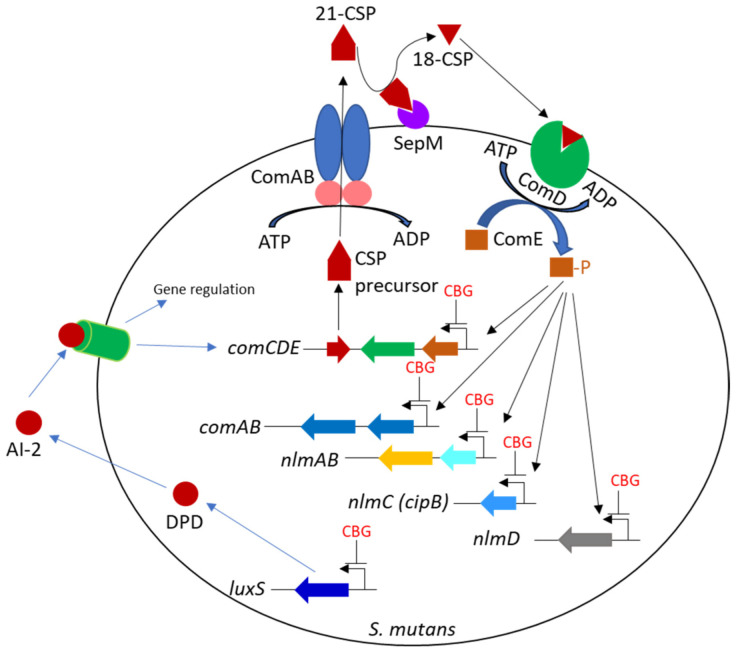
The anti-QS activity of CBG on *S. mutans*. CBG prevents the transcription of the *comC*, *comD*, *comE*, *comA*, *nlmA*, *nlmB*, *nlmC* and *luxS* genes, thus preventing the CSP and AI-2-induced QS signal transduction pathways.

*S. mutans* has several two- and three-component QS systems in which post-translationally modified oligopeptides are used as autoinducers [1,17,59]. One of these autoinducers is the 21-competence stimulating peptide (21-CSP) produced as a precursor by the *comC* gene, that during the export through the ABC ComAB transporter is cleaved into the mature 21-CSP [1,19]. 21-CSP can be further cleaved into 18-CSP by SepM [19,60] (Figure 11). The mature CSPs bind to the membrane ComD receptor leading to the induction of a phosphorelay with consequent phosphorylation and activation of the transcriptional regulator ComE [17]. Another family of autoinducers is the autoinducer-2 (AI-2) whose production relies on the *luxS* gene [61,62]. AI-2 is a chemical compound (furanosyl borate) formed from its precursor DPD (4,5-dihydroxy-2,3-pentanedione) [61,62]. AI-2 is acclaimed as a ‘universal autoinducer’ as it facilitates interspecies communication [63].

When we exposed the QS-mutant *S. mutans* strains (WT, *ΔluxS*, *ΔcomC*, *ΔcomE* and *ΔcomCDE*) to various CBG concentrations (0.75–5 µg/mL), different sensitivities to its anti-bacterial action were observed. Strains deficient in the ComCDE QS system showed increased sensitivity to CBG in comparison to the WT strain. This suggests that the anti-bacterial activity of CBG is affected by the ComCDE QS system. To further investigate the effect of the 21-CSP signal on the anti-bacterial effects of CBG, we incubated *S. mutans* WT and QS-knockout strains in the presence of CBG and 21-CSP. We observed that exogenously added 21-CSP prevented the anti-bacterial effect of CBG on the *ΔcomC* and *ΔcomE* strains, but not of the WT strain which constitutively expresses CSP. These findings suggest that 21-CSP induces a cell survival response that opposes the anti-bacterial action of CBG.

Since the *ΔcomE* mutant responded to the survival signal of 21-CSP, it is likely that the survival response induced by 21-CSP is mediated by a different pathway than the classical ComCDE cascade. Based on the finding that 21-CSP had no effect on the triple *ΔcomCDE* mutant, which differs from the *ΔcomE* and *ΔcomC* mutants by lacking the ComD receptor, we propose that the survival signal depends on the ComD receptor which propagates a downstream survival signaling pathway independent of ComE.

We next examined how CBG affects the early expression of genes regulated by the ComCDE QS system. We chose to follow the expression of the ComE-regulated *nlmA (SMU.150)*, *nlmB (SMU.151)* and *nlmC (cipB;SMU.1914)* genes, that encode for mutacins (bacteriocins) that affect autolysis and cell viability [64]. Following a 4 h incubation with 1.25 µg/mL CBG, there was a strong repression of the three *comCDE* genes as well as the ComE-regulated genes *nlmA-C* in all strains except for the WT. It should be noted that the *ΔluxS* strain responded to 1.25 µg/mL CBG with an 8 h delay in the log growth phase, and all the *comCDE* mutant strains showed growth arrest during the 20 h incubation period with this CBG concentration, while there was no growth inhibition on the WT strain. Thus, the reduced response of 1.25 µg/mL CBG on the tested gene expression in the WT strain goes along with its lower sensitivity to CBG. Another possibility is that there might be feedback mechanisms between the two QS systems, both systems being present in the WT, while only one of them in the mutant strains.

Since CBG down-regulated the *nlmA-C* genes also in the *ΔcomCDE* strain, the CBG-mediated gene repression seems to be independent of ComE, or it acts downstream of ComE. The addition of 21-CSP to the *S. mutans* strains significantly induced the expression of the *comCDE* genes (2-4-fold) and ComE-regulated genes (15-200-fold) in WT, *ΔluxS* and *ΔcomC* which have functional ComCDE systems, while, as expected, no response was observed in the *ΔcomE* and *ΔcomCDE* strains. CBG prevented the 21-CSP-induced expression of ComE-regulated genes in the WT, *ΔluxS* and *ΔcomC* strains, suggesting that CBG antagonizes the 21-CSP signal transduction pathway. An alternative explanation is that the CBG-mediated repression of the *nlmA-C* genes could not be overcome by 21-CSP. Since 21-CSP antagonized the anti-bacterial effects of CBG on *S. mutans* while the *nlmA-C* genes are still repressed by CBG in the presence of 21-CSP, it is likely that these genes are not involved in either the 21-CSP-induced survival or the anti-bacterial action of CBG.

The *comA* gene that is transcribed from a different operon than the *comCDE* operon [65] was also significantly upregulated by both 21-CSP and DPD. Its induction by DPD was dependent on the ComCDE QS system and strongly inhibited by CBG, again pointing to an anti-QS activity of CBG.

The susceptibility of WT and *ΔluxS* to the anti-bacterial activity of CBG was increased when DPD was added. However, this addition had no effect on the *ΔcomC* and *ΔcomE* strains, suggesting that also this effect depends on the ComCDE pathway. DPD had no anti-bacterial effect by its own. To our surprise, DPD inhibited the anti-bacterial activity of 1.25 and 0.75 µg/mL CBG on the *ΔcomCDE* strain. The opposite effect of DPD in the presence or absence of the ComCDE system indicates that a mild activation of the ComCDE pathway by AI-2 together with other effects induced by AI-2 promote the anti-bacterial effect of CBG, while in the absence of ComCDE, AI-2 provides pro-survival signals. It is known that AI-2 modulates the expression of an extensive range of genes in *S. mutans* [21]. Previous studies have shown that DPD reduces the sensitivity of a *ΔluxS Streptococcus intermedius* strain to antibiotics [66].

To get a better understanding of the AI-2-induced signals, we analyzed the expression level of *comCDE* genes, ComE-regulated genes and *luxS* in all our strains after a 4 h incubation with 1.25 µg/mL CBG and/or exogenous DPD. The expression of *comCDE* genes and ComE-regulated genes in WT, *ΔluxS* and *ΔcomE*, but not in *ΔcomC* and *ΔcomCDE*, were induced upon the addition of DPD, suggesting that AI-2 requires the 21-CSP encoded by *comC* for the induction of these genes. There has previously been a vague indication for a crosstalk between the AI-2 and ComCDE system, where an RNA sequencing analysis of a *ΔluxS Streptococcus suis* strain showed reduced expression of the CSP gene [67]. Our study supports that there is a crosstalk between these two QS systems.

It should be noted that the induction of *nlmA*, *nlmB* and *nlmC* (*cipB*) by AI-2 was milder (2.5-4.5-fold in WT) than that of 21-CSP (14-90-fold in WT), which is not surprisingly as the latter is the autoinducer of the ComCDE system in *S. mutans* [68]. In the absence of the ComCDE QS system, DPD repressed the expression of *nlmA*, *nlmB* and *nlmC* (*cibB*). The opposite effect of DPD on the gene expression in the *ΔcomCDE* strain in comparison to the WT and *ΔluxS* strain is reflected in bacterial survival in the presence of CBG. Notably, CBG prevented the DPD-mediated gene induction, indicating that CBG also antagonizes the AI-2 signals. CBG alone reduced *luxS* gene expression in all strains, suggesting a direct gene suppressive effect of CBG.

By using a bioluminescence assay based on the AI-1- and AI-2-deficient *V. harveyi* strains, the anti-QS effects of CBG were further proven. This was done by detecting the amount of AI-2 secreted by the various *S. mutans* strains in the presence or absence of CBG and/or 21-CSP. CBG inhibited AI-2 production in all strains tested, and the simultaneous presence of 21-CSP could not overcome this repression. It should be noted that CBG at 1–5 µg/mL inhibited the QS of *V. harveyi* that was related to CBG-induced LuxO expression and activity with a consequent downregulation in the LuxR gene [35]. In our bioassay, the *S. mutans* condition medium was diluted 1:10, resulting in a final CBG concentration of 0.125 µg/mL which is below that required for the anti-QS effect on *V. harveyi*.

**Figure 12:**
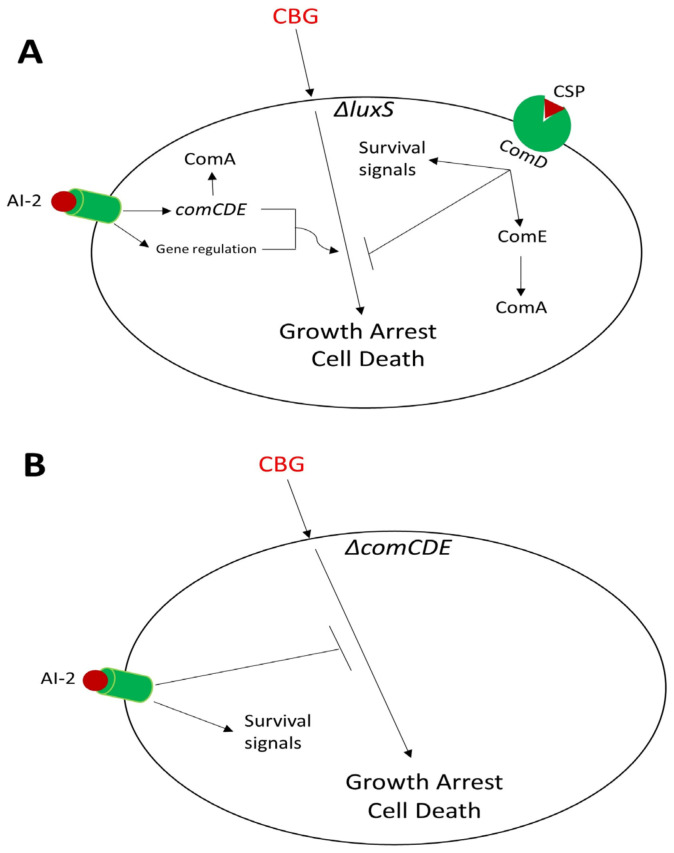
(**A**,**B**). The prevention and promotion of the anti-bacterial effect of CBG by the autoinducers 21-CSP and AI-2 in *ΔluxS* (**A**) and *ΔcomCDE* (**B**) *S. mutant* strains. In the *ΔluxS* strain, 21-CSP prevents, while AI-2 enhances the anti-bacterial effect of CBG. In the *ΔcomCDE* strain, AI-2 prevents the anti-bacterial effect of CBG.

In summary, the present study shows that the two QS systems LuxS and ComCDE affect the susceptibility of *S. mutans* to CBG, and CBG acts as an anti-quorum sensing compound that represses the expression of *comCDE*, *luxS* and ComE-regulated genes (Figure 11 and Figure 12). We have further provided evidence that there is a crosstalk between the AI-2 and ComCDE QS systems.

## Data Availability

Raw data are available upon reasonable request.

## Supplementary Materials

The following supporting information can be downloaded at https://www.mdpi.com/article/10.3390/biomedicines11030668/s1.
