# Supplementary material for: The Antibacterial Effect of Cannabigerol toward Streptococcus mutans Is Influenced by the Autoinducers 21-CSP and AI-2
Source: Biomedicines. 2023 Feb 22;11(3):668. doi: 10.3390/biomedicines11030668 (PMC10045765; doi:10.3390/biomedicines11030668)
Supplement: Supplementary file 1 [file biomedicines-11-00668-s001.zip › biomedicines-2219545-supplementary.pdf]

Supplementary Table S1. Primers used for real-time PCR.

	Forward Primer	Reverse Primer
16S rRNA	CCTACGGGAGGCAGCAGTAG	CAACAGAGCTTTAGATCCGAAA
comA	ACGAGCCTAACAAGGGGATT	CCCTGAGGCATTTGTTCAAT
comC	GACTGATGAATTAGAGATTATCATTGG	TTTCCCAAAGCTTGTGTAAAACT
comD	TGAAAATAGCATAGGTGAG TCAAAG	ATTTAGGTTAGCTGATTAACACTATAC AC
comE	CACAACAACTTATTGACGCTATCCC	TGATTGGCTACTTCCAGTCCTTTC
nlmA	AATGGACAGCCAAACACTTTC	TAACAAGAGTCGCACCTGCC
nlmB	TGTCAGAAGTTTTTGGTGG	ACTCCAGCACATCCAGCAAG
nlmC	TTGTGCAGCAGGTATTGCTC	AAGAGCTCCTCCGATTCCTC
luxS	ACTGTTCCCCTTTTGGCTGTC	AACTTGCTTTGATGACTGTGGC

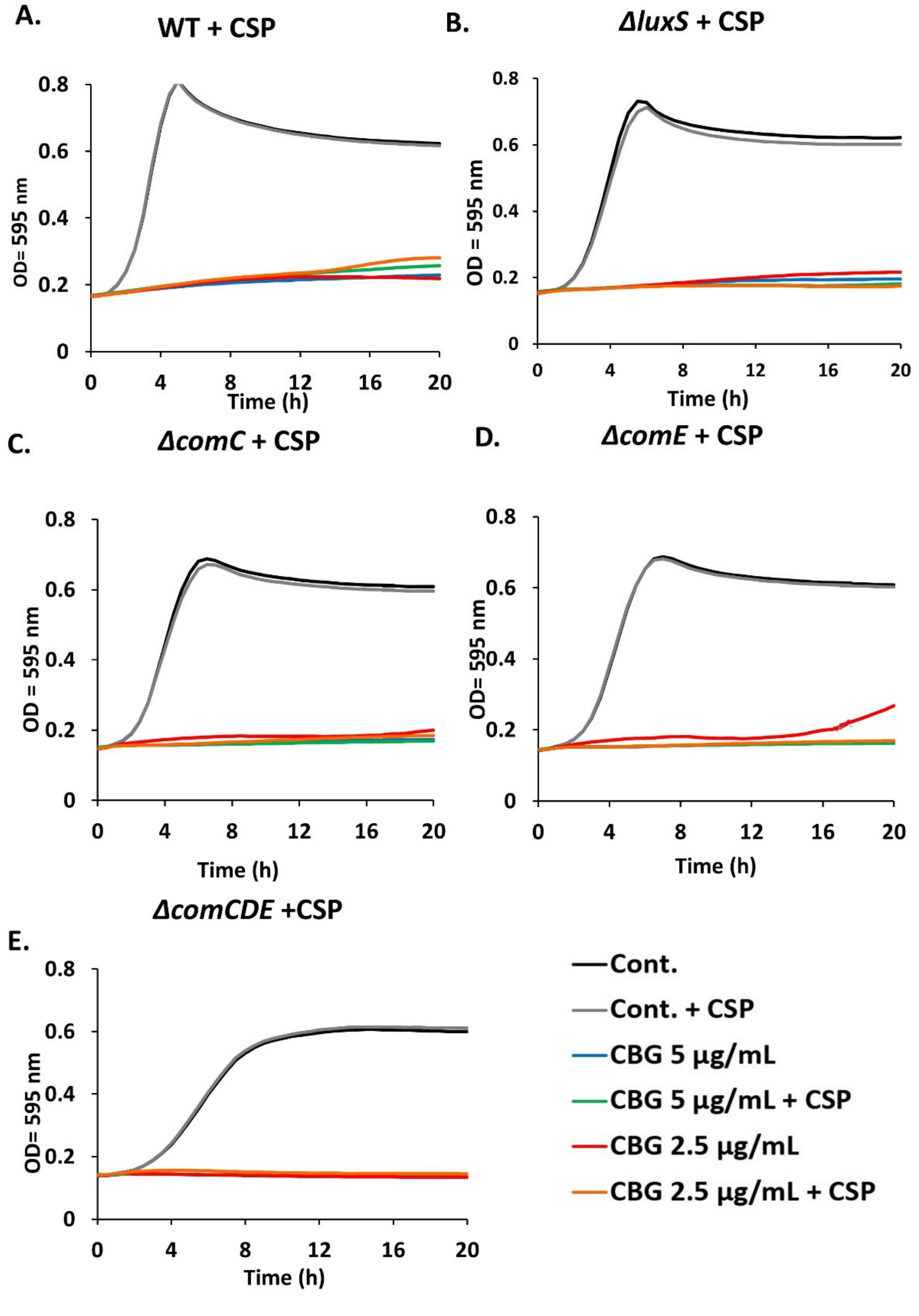

Supplementary Figure S1: 21-CSP did not prevent the anti-bacterial effect of 2.5 and 5 µg/mL CBG on the various *S. mutans* strains. A-E. Kinetic studies of the planktonic growth of *S. mutans* incubated in the absence or presence of CBG (2.5 or 5 µg/mL) with or without 21-CSP (1 µg/mL) with an initial OD_{600nm} of 0.1. **A.** WT; **B.** *ΔluxS*; **C.** *ΔcomC*; **D.** *ΔcomE*; and **E.** *ΔcomCDE*. *n* = 3.

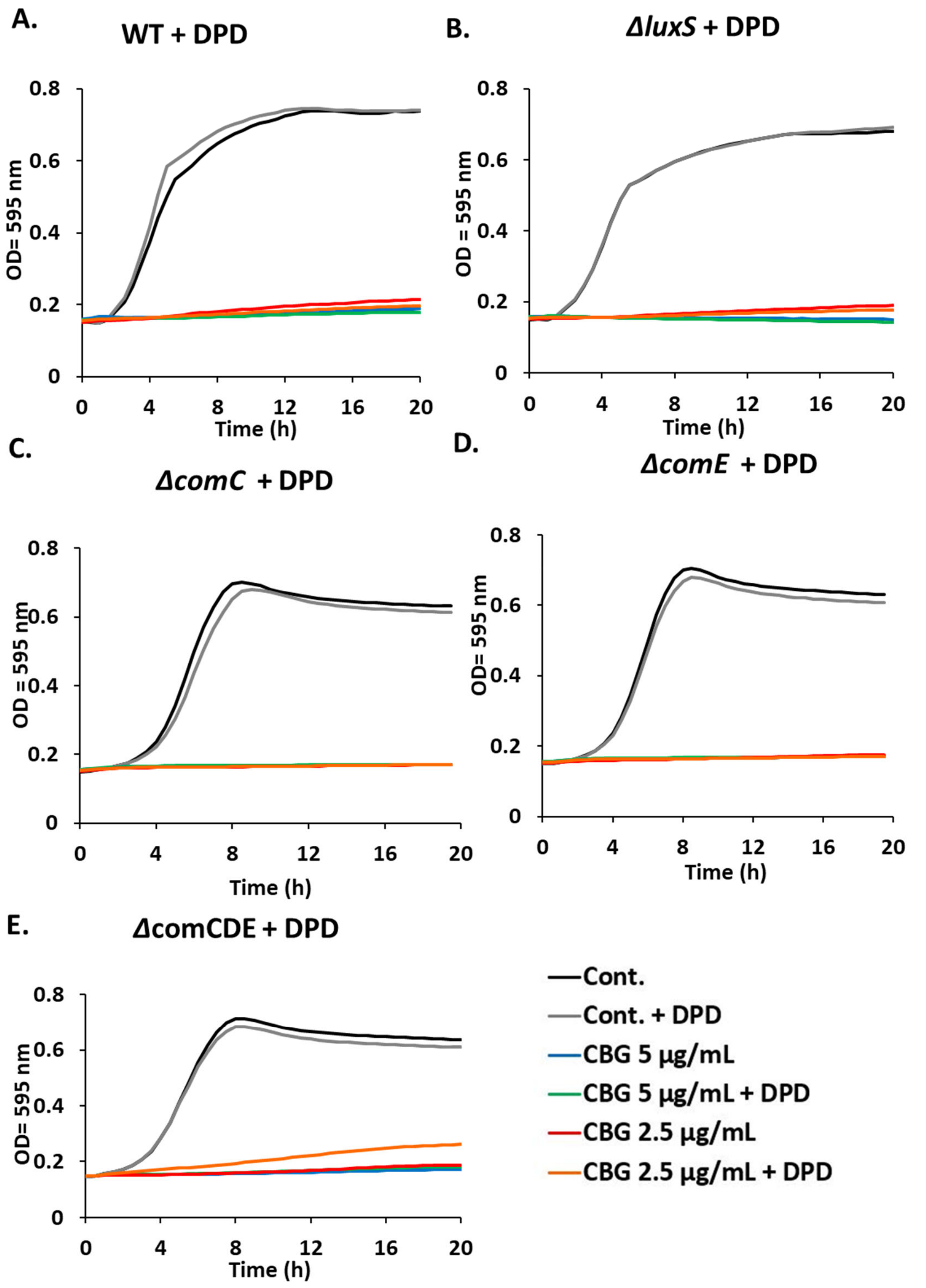

Supplementary Figure S2: AI-2 did not prevent the anti-bacterial effect of CBG of *S. mutans* strains at higher concentrations. A-E. Kinetic studies of the planktonic growth of *S. mutans* incubated in the absence or presence of CBG (2.5 or 5 µg/mL) with or without DPD (pre-AI-2) (5 µM) with an initial OD_{600nm} of 0.1. **A.** WT; **B.** *ΔluxS*; **C.** *ΔcomC*; **D.** *ΔcomE*; and **E.** *ΔcomCDE*. *n* = 3.

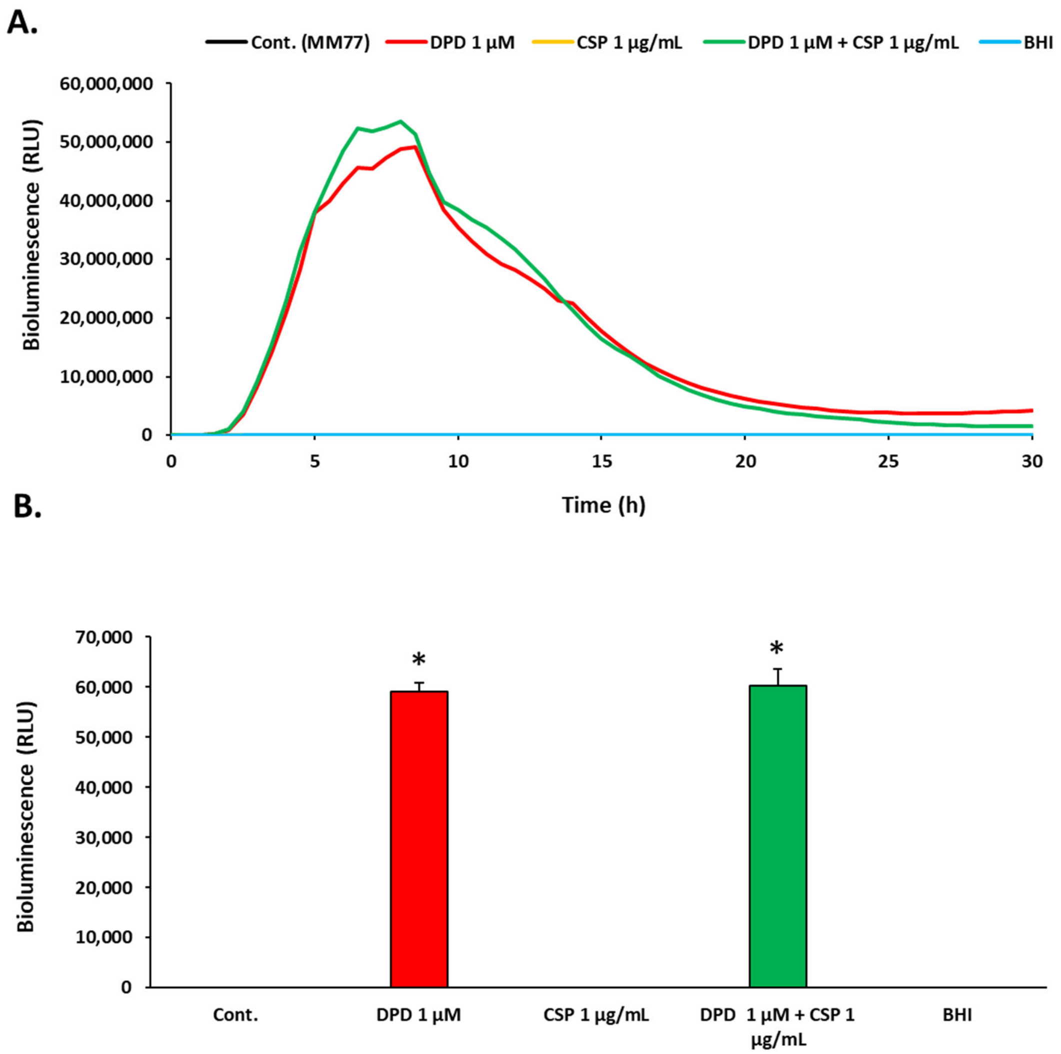

Supplementary Figure S3: The relative bioluminescence of *Vibrio harveyi* strain MM77 incubated in the absence or presence of 1 µM DPD and/or 1 µg/mL 21-CSP was measured for 30 h. The bioluminescence was corrected for differences in bacterial growth by simultaneously measuring the optical density at 595 nm. *n* = 3. **B.** The relative bioluminescence as determined by the area under the curve (AUC) of the graph presented in **A.** *n* = 3**.**

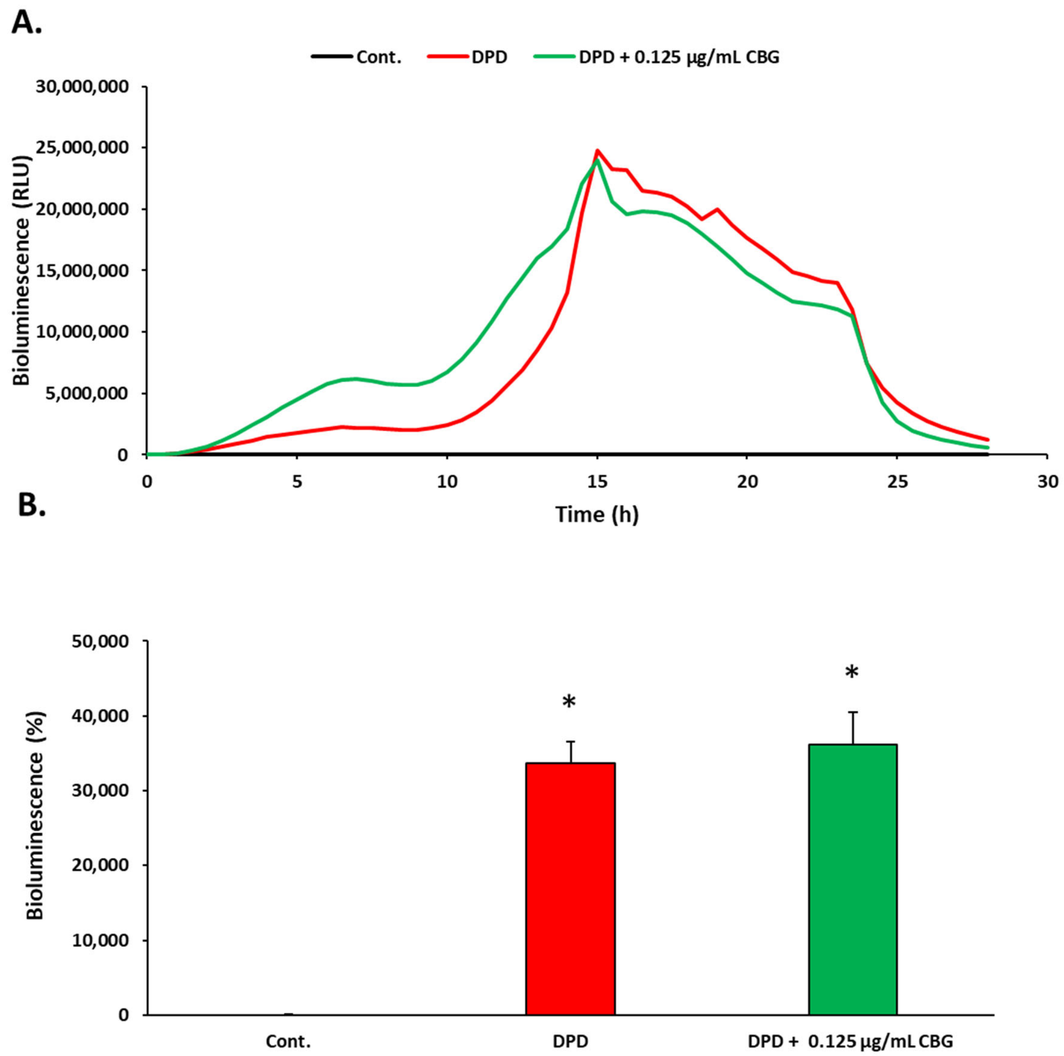

Supplementary Figure S4: The relative bioluminescence of *Vibrio harveyi* strain MM77 incubated in the absence or presence of 1 µM DPD and/or 1 µM DPD + 0.125 µg/mL CBG was measured for 30 h. The bioluminescence was corrected for differences in bacterial growth by simultaneously measuring the optical density at 595 nm. $n = 3$. **B.** The relative bioluminescence as determined by the area under the curve (AUC) of the graph presented in **A.** $n = 3$.
